# Lower mean platelet volume predicts poor prognosis in renal cell carcinoma

**DOI:** 10.1038/s41598-017-07168-x

**Published:** 2017-07-27

**Authors:** Zhi-yuan Yun, Xin Zhang, Yan-song Liu, Tiemin Liu, Zhi-ping Liu, Rui-tao Wang, Kai-jiang Yu

**Affiliations:** 1 Department of Internal Medicine, Harbin Medical University Cancer Hospital, Harbin Medical University, Harbin, Heilongjiang 150081 China; 2 Department of Intensive Care Unit, Harbin Medical University Cancer Hospital, Harbin Medical University, Harbin, Heilongjiang 150081 China; 3 Division of Hypothalamic Research, Department of Internal Medicine, UT Southwestern Medical Center, Dallas, TX 75390 USA; 4 Departments of Internal Medicine and Molecular Biology, University of Texas Southwestern Medical Center, Dallas, TX USA

## Abstract

Altered mean platelet volume (MPV) is found in several malignancies. Remarkably, there is little consensus on using the value of MPV in the prognostic evaluations of renal cell carcinoma (RCC). The aim of this study is to examine the feasibility of MPV value as a prognostic indicator of RCC. The retrospective study recruited 306 consecutive RCC patients between January 2009 and December 2009. The relationships between MPV and clinicopathological characteristics were analyzed. Kaplan-Meier method and Cox regression were used to evaluate the prognostic impact of MPV. Of the 306 RCC patients, low MPV levels were detected in 61 (19.9%) patients. Reduced MPV was associated with histology types, T classification, UCLA Integrated Scoring System (UISS) category, and Mayo clinic stage, size, grade, and necrosis score (SSIGN) category (P < 0.05). Patients with decreased MPV had significantly shorter survival time than patients with normal MPV (P < 0.001). Cox regression analysis revealed that reduced MPV was an independent prognostic factor for overall survival (hazard ratio, 1.758; 95% confidence interval [CI], 1.083–2.855, P = 0.023). Moreover, the prognostic accuracy of TNM stage, UISS, and SSIGN prognostic models were improved when MPV was added. In conclusion, reduced MPV is identified as an independent predictor of adverse clinical outcome in RCC.

## Introduction

Renal cell carcinoma (RCC) is the most common type of malignant tumor in the adult kidney and represents 2–3% of all adult cancers^1^. Despite increasing insights into the biology of RCC and advances in therapeutic techniques to RCC, one-third of RCC patients present with metastasis at the time of diagnosis. Therefore, searching for biomarkers to predict the prognosis in RCC is of critical importance.

Platelets are critical for cancer progression and metastasis^2^. The interplay between platelets and tumor cells lead to tumor growth, angiogenesis, and dissemination^3^. Elevated platelets are associated with a reduction in overall survival and poorer prognosis in various types of cancer, such as pancreatic, gastric, colorectal, endometrial, and ovarian cancers^4–8^. However, platelet count is not only determined by the rate of production but also determined by how much platelets are used in the body. A normal platelet count could conceal the presence of highly hypercoagulative and pro-inflammatory cancer phenotypes in the presence of efficient compensatory mechanisms^9^.

Mean platelet volume (MPV), the most commonly used index of platelet size, is a surrogate marker of platelet activation^10^. Altered MPV levels were observed in gastric cancer, ovarian cancer, lung cancer, colon cancer, and breast cancer^11–14^. However, its clinical implications in RCC have not been reported.

The purpose of this study was to assess whether MPV holds a prognostic role in RCC patients.

## Results

306 RCC patients were enrolled in this study between Jan 2009 and Dec 2009. The median age was 57.8 ± 8.5 years (range 37–80) with 196 men and 110 women. Of the 306 patients, 286 presented with locally confined disease (T1–2), while 20 presented with locally advanced disease (T3–4). Of the 306 patients, 290 had no metastasis (M0), and 16 presented with metastasis (M1). 40 of the 306 cases were categorized as stage I and stage II, 266 as stage III and stage IV. With a median follow up of 60 months, 100 (32.7%) patients had death events.

**Figure 1:**
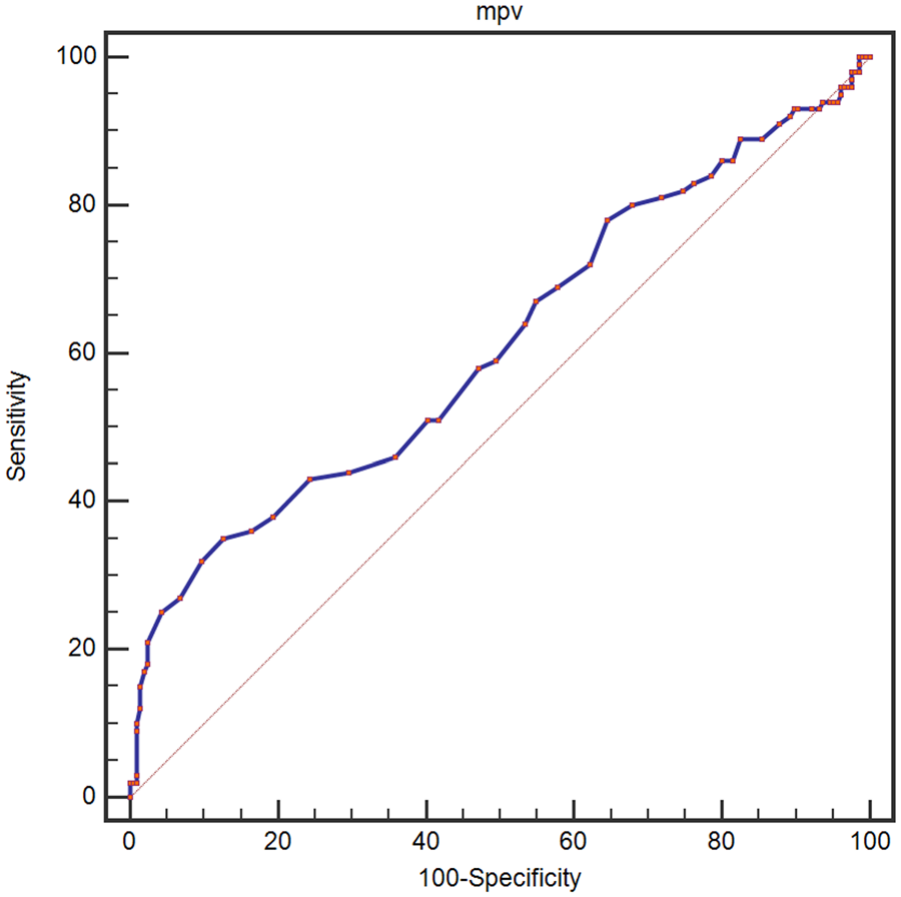
Optimized cut-off was determined for MPV using standard ROC curve analysis.

A ROC curve for OS prediction was plotted to verify the optimal cut-off value for MPV, which was 7.5 fL (Fig. 1). It demonstrated that MPV predicts cancer prognosis with a sensitivity of 35.0% and a specificity of 87.4% (AUC = 0.610, 95% CI: 0.553–0.665, p = 0.002). Patients then were sub-divided into 2 groups: patients with MPV ≤ 7.5 fL and patients with MPV > 7.5 fL. There were 61 (19.9%) patients with MPV ≤ 7.5 fL and 245 (80.1%) patients with MPV > 7.5 fL.

The relationship between MPV and clinical characteristics is shown in Table 1 and Table 2. Our study revealed that MPV was associated with histology types, T classification, UISS category, and SSIGN category. However, no significant differences were found between the groups with regard to age, gender, tumor size, Fuhrman grade, microvascular invasion, lymph node metastasis, distant metastasis, ECOG PS, and tumor stage.

**Table 1:** Baseline characteristics of patients with renal cell carcinoma according to MPV levels.

Variables	Total	MPV ≤ 7.5	MPV > 7.5	P value
	n (%)	n (%)	n (%)	
Age (years)				0.396
<55	125 (40.8)	22 (36.1)	103 (42.0)	
≥55	181 (59.2)	39 (63.9)	142 (58.0)	
Gender				0.782
Male	196 (64.1)	40 (65.6)	156 (63.7)	
Female	110 (35.9)	21 (34.4)	89 (36.3)	
Tumor size (cm)				0.396
≤4.0	125 (40.8)	22 (36.1)	103 (42.0)	
>4.0	181 (59.2)	39 (63.9)	142 (58.0)	
Histology				<0.001
Clear cell	279 (91.2)	57 (93.4)	222 (90.6)	
Papillary	15 (4.9)	2 (3.3)	13 (5.3)	
Chromophobe	9 (2.9)	2 (3.3)	7 (2.9)	
Others	3 (1.0)	0 (0)	3 (1.2)	
Fuhrman grade				0.443
G1+G2	203 (66.3)	43 (70.5)	160 (65.3)	
G3+G4	103 (33.7)	18 (29.5)	85 (34.7)	
T classification				0.020
T1+T2	286 (93.5)	53 (86.9)	233 (95.1)	
T3+T4	20 (6.5)	8 (13.1)	12 (4.9)	
Lymph node metastasis				0.165
Absent	295 (96.4)	57 (93.4)	238 (97.1)	
Present	11 (3.6)	4 (6.6)	7 (2.9)	
Distant metastasis				0.602
Absent	290 (94.8)	57 (93.4)	233 (95.1)	
Present	16 (5.2)	4 (6.6)	12 (4.9)	
TNM stage				0.216
I/II	266 (86.9)	48 (78.7)	218 (89.0)	
III/IV	40 (13.1)	13 (21.3)	27 (11.0)	
Microvascular invasion				0.631
Absent	257 (84.0)	50 (82.0)	207 (84.5)	
Present	49 (16.0)	11 (18.0)	38 (15.5)	
ECOG PS				0.188
0	262 (85.6)	49 (80.3)	213 (86.9)	
≥1	44 (14.4)	12 (19.7)	32 (13.1)	
UISS category				<0.001
Low risk	129 (42.2)	20 (32.8)	109 (44.5)	
Mediate risk	148 (48.4)	36 (59.0)	112 (45.7)	
High risk	29 (9.5)	5 (8.2)	24 (9.8)	
SSIGN category				<0.001
0–3	232 (75.8)	40 (65.6)	192 (78.4)	
4–7	68 (22.2)	20 (32.8)	48 (19.6)	
≥8	6 (2.0)	1 (1.6)	5 (2.0)

**Table 2:** Baseline characteristics of patients with renal cell carcinoma according to MPV levels.

Variables	MPV ≤ 7.5	MPV > 7.5	P value
Age (years)	57.9 (11.4)	55.9 (11.1)	0.214
Gender (male, %)	40 (65.6)	156 (63.7)	0.782
Smoker (n, %)	10 (16.4)	26 (10.6)	0.210
Drinking (n, %)	5 (8.2)	11 (4.5)	0.245
BMI (kg/m^2^)	24.3(3.0)	24.3 (3.9)	0.928
FPG (mmol/L)	5.00 (4.65–5.60)	5.17 (4.80–6.00)	0.080
WBC (×10^9^/L)	6.55 (1.40)	6.46 (2.13)	0.738
Hemoglobin (g/dl)	122.0 (26.7)	136.5 (16.9)	<0.001
Platelet count (×10^9^/L)	318.6 (120.2)	223.5 (71.1)	<0.001

Data are expressed as means (SD) or median (IQR). FPG, fasting plasma glucose; WBC, white blood cell; MPV, mean platelet volume. Abbreviations: see to Table 1.

**Figure 2:**
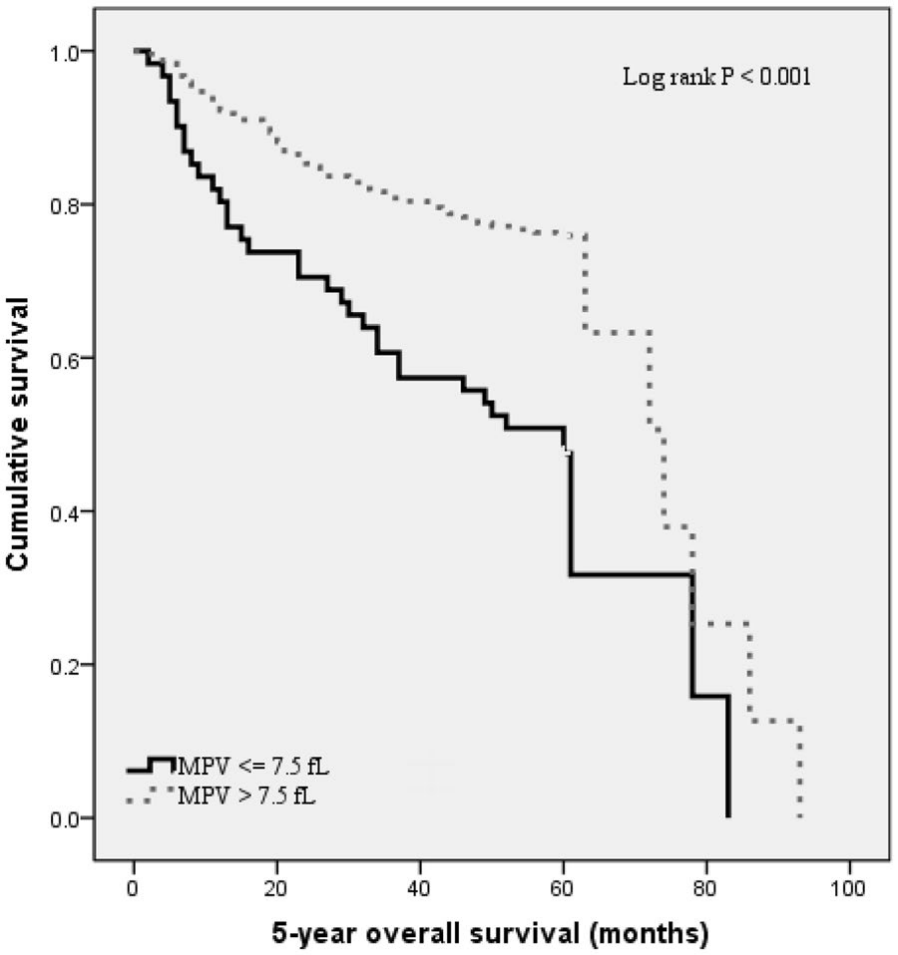
Kaplan–Meier analysis of overall survival in RCC patients.

Kaplan-Meier survival analysis was performed to compare OS according to MPV levels. Patients with low MPV levels had worse 5-year OS (50.8% vs. 75.9%) than those with high MPV levels as is shown in Fig. 2 (P < 0.001).

To further determine the prognostic value of MPV, we applied univariate and multivariate Cox proportional hazard models to evaluate the hazard ratio (HR) and 95% confidence interval (CI). In the univariate analyses, MPV together with age (categorical variable), hemoglobin, platelet count, tumor size, T classification, lymph node metastasis, distant metastasis, microvascular invasion, and TNM stage were associated with OS (Table 3). Variables with p value lower than 0.10 in univariate analysis were included in the multivariate analysis. In the multivariate analyses, MPV (categorical variable), hemoglobin, platelet count, tumor size, lymph node metastasis, and TNM stage were independent factors for OS (Table 4). Patients with MPV ≤ 7.5 fL had a HR of 1.758 [95% CI: 1.083–2.855, P = 0.023] for OS.

**Table 3:** Univariate analysis of overall survival in patients with renal cell carcinoma.

	Hazard ratio	95% CI	*P*-value
Age (years) (≥60 versus <60)	1.552	1.018–2.366	0.041
Gender (male versus female)	1.287	0.836–1.981	0.251
Smoker (yes versus no)	1.400	0.793–2.472	0.246
Drinking (yes versus no)	0.789	0.290–2.149	0.643
BMI (kg/m^2^)	0.956	0.899–1.017	0.150
FPG (mmol/L)	1.081	0.969–1.207	0.162
WBC (×10^9^/L)	1.061	0.975–1.154	0.170
Hemoglobin (g/dl)	0.977	0.968–0.986	<0.001
Platelet count (×10^9^/L)	1.006	1.004–1.007	<0.001
MPV (fL) (≤7.5 versus >7.5)	2.513	1.657–3.812	<0.001
Tumor size (cm)			
(>4.0 versus ≤4.0)	2.312	1.457–3.669	<0.001
Histology			
(Others versus Clear cell)	2.300	0.844–6.268	0.103
Fuhrman grade			
(G3+G4 versus G1+G2)	1.163	0.759–1.781	0.489
T classification			
(T3+T4 versus T1+T2)	4.543	2.596–7.950	<0.001
Lymph node metastasis			
(Present versus Absent)	4.792	2.802–8.194	<0.001
Distant metastasis			
(Present versus Absent)	2.659	1.335–5.296	0.005
Microvascular invasion			
(Present versus Absent)	2.357	1.477–3.762	<0.001
TNM stage			
(III/IV versus I/II)	4.985	3.193–7.782	<0.001
ECOG PS			
(≥1 versus 0)	2.078	1.303–3.315	0.002

Abbreviations: see to Table 1 and Table 2.

**Table 4:** Multivariate analysis of overall survival in patients with renal cell carcinoma.

	Hazard ratio	95% CI	*P*-value
Age (years) (≥60 versus <60)	1.155	0.739–1.804	0.528
Hemoglobin (g/dl)	0.986	0.977–0.996	0.006
Platelet count (×10^9^/L)	1.003	1.001–1.006	0.005
MPV (fL) (≤7.5 versus >7.5)	1.758	1.083–2.855	0.023
Tumor size (cm)			
(>4.0 versus ≤4.0)	1.867	1.134–3.075	0.014
T classification			
(T3+T4 versus T1+T2)	0.788	0.320–1.942	0.605
Lymph node metastasis			
(Present versus Absent)	2.539	1.299–4.961	0.006
Distant metastasis			
(Present versus Absent)	0.595	0.252–1.404	0.236
Microvascular invasion			
(Present versus Absent)	1.193	0.649–2.190	0.570
TNM stage			
(III/IV versus I/II)	4.034	1.911–8.516	<0.001

Variables that showed a p-value < 0.10 in univariate analysis were included in a multivariate Cox proportional hazards regression model. CI, confidence interval. Abbreviations: see to Table 1 and Table 2.

To further confirm the predictive ability of MPV, we compared MPV with classic prognostic models, such as TNM staging system, the University of Los Angeles integrated staging system (UISS) scoring systems, and the Mayo clinic stage, size, grade, and necrosis score (SSIGN), respectively. Concordance index (C-index) and Akaike information criteria (AIC) analysis were used for prognostic power evaluation. As shown in Table 5, the C-indexes for OS were 0.618, 0.631, and 0.699, respectively, when assessed with the TNM, UISS, and SSIGN models alone. The C-indexes were improved to 0.700, 0.681, and 0.734, respectively, when MPV was added into models. Moreover, the AIC value of each model combined with MPV was lower than each model alone.

**Table 5:** Comparison of the prognostic accuracy of the prognostic models and MPV levels.

Model	Overall Survival	
	C-Index	AIC
MPV	0.610	756.662
TNM stage	0.618	713.406
TNM stage + MPV	0.700	695.754
UISS	0.631	734.622
UISS + MPV	0.681	721.262
SSIGN	0.699	671.708
SSIGN + MPV	0.734	662.336

MPV = Mean platelet volume, C-index = Harrell concordance index, AIC = Akaike information criteria, TNM stage = tumor, node and metastasis stage, SSIGN = the Mayo clinic stage, size, grade, and necrosis score, UISS = University of Los Angeles integrated staging system.

## Discussion

This study found that MPV correlates with patient’s survival and is an independent predictor of adverse clinical outcome in RCC.

Platelets act as a crucial modulator in tumor development, tumor cell growth, angiogenesis, and metastasis. In RCC, platelet-derived growth factor-alpha alpha receptor over-expression is associated with adverse outcomes^15^. High platelet-derived endothelial cell growth factor activity is linked to the malignant potential of RCC^16^. Low expression of platelet-derived growth factor-BB (PDGF-BB) predicts distant metastasis and poor prognosis^17^. On the contrary, blocking platelet-derived growth factor-D/platelet-derived growth factor receptor beta signaling inhibits the progression of metastatic RCC^18^. However, these parameters reflecting activated platelets are expensive and are not commonly used in clinical practice. MPV is available in routine practice and can be easily evaluated prior to treatment.

The mechanisms underlying the association between MPV and survival in RCC have yet to be fully defined. There are several mechanisms that are potentially responsible for decreased MPV in RCC. Firstly, chronic inflammation plays a pivotal role. There is a firm linkage between inflammation and cancer^19^. MPV was an early marker of activated platelets. Decreased MPV could be regarded as an enhanced consumption of large platelets in inflammatory states^10^. Recent studies confirmed that low levels of MPV are associated with high-grade inflammatory diseases and are reversed in the course of anti-inflammatory therapy^10^. Secondly, platelets can induce tumor growth via increasing angiogenesis. Platelets coordinate complex angiogenic responses through a number of different mechanisms, including direct cellular contact, local release of pro-angiogenic proteins into the tumor microenvironment, and recruitment of distant cells into the tumor mircroenvironment^20^. Thirdly, platelets promote tumor cell migration and invasion. A platelet cloak protects tumor cells in circulation from extreme shear forces encountered in the vascular system, preventing mechanical damage to the cells^21^. Moreover, platelets release transforming growth factor-β1 (TGF-β1) that induces phenotypic changes of epithelial to mesenchymal-like transition of tumor cells and facilitates their extravasation to distant sites during metastasis. Activated platelets release secretory factors that promote chemokines, proteolytic enzymes and microparticles within the microenvironment to promote tumor cell invasion^22^.

In line with our results, Aksoy *et al*. showed that solid tumors with bone marrow metastasis were more likely to have low MPV levels^23^. Inagaki N and Kumagai S *et al*. revealed that low MPV level was associated with poor prognosis in non-small-cell lung cancer^13, 24^. These data are also consistent with the current knowledge that anti-platelet treatment is considered to be a part of cancer adjuvant therapy^3^. Therefore, our results may help to inform treatment decisions and predict treatment outcomes.

The present study has some limitations. First, this was a single-center retrospective study and additional larger validation studies with multiethnic groups are needed to confirm our results. Second, we were unable to explore the exact mechanism of MPV in RCC. Third, the patients were composed of Chinese. The application to other ethnic groups still needs further investigation.

In conclusion, MPV is easily available with routine blood counts. Reduced MPV may serve as a marker of adverse prognosis in RCC. Further studies are warranted to clarify the exact role of MPV in RCC.

## Patients and Methods

### Study population

This study consisted of 306 consecutive RCC cases (mean age 56.3 ± 11.1 years, range 19–80 years). Cases were admitted to Harbin Medical University Cancer Hospital, Harbin Medical University between January 2009 and December 2009. All patients underwent complete surgical resection. The pathologic diagnoses of RCC were evaluated by pathologists from biopsy reports. None of the patients received preoperative chemotherapy or radiation therapy. Patients were excluded if they had hematological disorders, coronary artery  disease, hypertension, diabetes mellitus, and medical treatment with anticoagulant, statins, and acetylic salicylic acid.

Standard demographic and clinicopathological data were collected from the patients’ records in hospital. Survival data were obtained through follow-up. Overall survival (OS) was defined as the interval from the date of diagnosis to death or last follow-up. The median follow-up time was 60 months. White blood cell (WBC), haemoglobin, and platelet indices were measured by an autoanalyzer (Sysmex XE-2100, Kobe, Japan). The whole blood samples were collected in EDTA-containing tubes, and all samples were processed within 30 minutes after blood collection. The method was validated in a previous report^25^.

The Institutional Ethics Review Board of Harbin Medical University Cancer Hospital approved this study prior to commencement of data collection and waived the informed consent requirement because it was a retrospective study. All studies were conducted according to guidelines (Declaration of Helsinki) for biomedical research.

### Statistical analysis

All statistical analyses were performed using SPSS Statistics version 22.0 (SPSS Inc., Chicago, IL, USA). The descriptive statistics were presented as means ± SD or medians (interquartile range) for continuous variables and percentages of the number for categorical variables. Inter-group differences in categorical variables were assessed for significance using the Chi-square test; differences in continuous variables were assessed using the Mann-Whitney U test or t-test. OS was analyzed using the Kaplan-Meier method, and differences between curves were assessed for significance using the log-rank test. Variables that showed a p value < 0.1 in univariate analysis were included in a multivariate Cox proportional hazards regression model. Multivariate Cox proportional hazards modeling was used to identify independent prognostic factors. Receiver-operating characteristics (ROC) curve analysis was performed to identify cut-off value of MPV. A value of p < 0.05 was regarded as a significant difference between groups.
